# Whole-genome-based characterization of Escherichia albertii strains isolated from paediatric diarrhoeal cases in Kolkata, India

**DOI:** 10.1099/mgen.0.001363

**Published:** 2025-04-08

**Authors:** Goutam Chowdhury, Yuki Hoshiko, Miki Okuno, Kei Kitahara, M John Albert, Shin-ichi Miyoshi, Yoshitoshi Ogura, Shanta Dutta, Thandavarayan Ramamurthy, Asish K. Mukhopadhyay

**Affiliations:** 1 Division of Bacteriology, ICMR-National Institute of Cholera and Enteric Diseases, Kolkata, India; 2 Collaborative Research Centre of Okayama University for Infectious Diseases, ICMR-National Institute of Cholera and Enteric Diseases, Kolkata, India; 3 Division of Microbiology, Department of Infectious Medicine, School of Medicine, Kurume University, Fukuoka, Japan; 4 Department of Microbiology, College of Medicine, Kuwait University, Jabriya, Kuwait; 5 Graduate School of Medicine, Dentistry and Pharmaceutical Sciences, Okayama University, Okayama, Japan

**Keywords:** antimicrobial resistance, diarrhoea, *E. albertii*, virulence, whole-genome sequence

## Abstract

*Escherichia albertii* is a Gram-negative facultative anaerobic bacterium that causes diarrhoea in humans. This study shows the isolation of *E. albertii* from hospitalized paediatric diarrhoeal cases and genome-based characteristics with putative virulence factors and antimicrobial resistance. *E. albertii* isolates were identified by species-specific PCR, targeting the gene encoding cytolethal distending toxin (*Ea-cdt*). The genome of *E. albertii* was sequenced to identify (i) genes encoding virulence factors (ii) antibiotic resistance-encoding genes, including the mobile genetic elements and (iii) core gene-based phylogenetic relationships and pan-genome features. A total of 10 (1.2%) *E. albertii* isolates were isolated from 854 faecal samples, of which 6 (60%) were found as the sole pathogen and the remaining 4 (40%) were identified along with other pathogens, such as enteroaggregative *Escherichia coli*, rotavirus and adenovirus. Patients from whom *E. albertii* was isolated presented cholera-like diarrhoea, i.e. with watery stool (60%) with moderate dehydration (100%), fever (20%) and abdominal pain (20%). The antimicrobial susceptibility testing of *E. albertii* showed that most of the isolates were susceptible or reduced susceptible to most of the antibiotics except resistance to erythromycin (80%), tetracycline (50%), nalidixic acid (40%), ampicillin (40%), doxycycline (30%) and ceftriaxone (20%). In the whole-genome sequence, *E. albertii* isolates revealed several virulence-encoding genes, namely the intimin (*eae*, *E. coli* attaching and effacing), the cytolethal distending toxin type II subunit A (*cdt-IIA*), adhesion (*paa*, porcine attaching- and effacing-associated), non-LEE (locus of enterocyte effacement) encoded effector A (*nleA*) and antimicrobial resistance genes (ARGs) conferring resistance to tetracycline (*tetA*, *tetR*), sulphonamides (*sul2*), fluoroquinolones (*qnrS*) and beta-lactamases (*bla*_CTX-M_, *bl*a_TEM_). The SNP-based phylogenetic analysis of 647 whole genomes of *E. albertii* isolates from the National Center for Biotechnology Information databases did not reveal any comparable clustering pattern based on the biological source and place of isolation. The genome of some of the *E. albertii* was closely related to those of the isolates from China and the United Kingdom. The PFGE patterns revealed that most of the *E. albertii* isolates were distinct clones. This study reports on the extensive genome analysis of diarrhoea-associated *E. albertii* harbouring multiple virulence and ARGs.

­

**Impact Statement**

*Escherichia albertii* has been recently designated as one of the causative agents of diarrhoea in humans. We have identified ten *E. albertii* isolates from hospitalized paediatric diarrhoeal cases, of which six were sole pathogens. Most of the cases had watery diarrhoea with moderate dehydration. The antimicrobial susceptibility testing of *E. albertii* isolates showed resistance to erythromycin, tetracycline, nalidixic acid and ampicillin. Whole-genome sequence analysis has shown several virulence genes that encode intimin (*Escherichia coli* attaching and effacing), the cytolethal distending toxin type II subunit A, adhesion (porcine attaching- and effacing-associated) and non-LEE (locus of enterocyte effacement) encoded effector A. The single nucleotide polymorphism-based phylogenetic analysis of *E. albertii* isolates did not reveal any comparable clustering pattern with the sequences existing in the NCBI database, and the tested isolates were distinct in the pulsed-field gel electrophoresis analysis. The clinical and epidemiological importance of *E. albertii* needs further detailed study.

## Data Summary

All the sequence data generated in this study have been submitted to the NCBI BioProject database under accession number PRJNA975620, and the strains finally used in this study are listed in Table S1available in the online Supplementary Material.

## Introduction

*Escherichia albertii* is a recently recognized member of the genus *Escherichia* and an emerging zoonotic/foodborne enteropathogenic bacterium that causes watery diarrhoea, abdominal distention, fever and vomiting in humans [1]. *E. albertii* is a close relative of *Escherichia coli* and has been frequently misidentified as *Hafnia alvei,* enteropathogenic *E. coli* (EPEC), enterohaemorrhagic *E. coli* (EHEC) and *Shigella boydii* serotype 13 due to their similarity in phenotypic and genetic features [2,3]. Presently, *E. albertii* has been included as a novel species of the genus *Escherichia* and named as *E. albertii* [4]. *E. albertii* has been reported in Bangladesh [5], Brazil [6], China [7], Japan [8], Mexico [9], Poland [10], Switzerland [11], Great Britain [12] and the USA [13] from sporadic diarrhoea and outbreaks of diarrhoea in humans. *E. albertii* has also been identified from wild and domestic birds, cats, dogs, pigs, seals and raccoons in different countries [14,16], but its definite reservoir(s), transmission routes and clinical significance have not yet been fully established.

*E. albertii* infections in humans exhibit several signs and symptoms, such as watery diarrhoea, abdominal pain, vomiting, dehydration and high fever in some cases [17]. Several recent studies have demonstrated that *E. albertii* was the etiologic agent in multiple diarrhoeal outbreaks, most of which had previously been incorrectly identified as EPEC and EHEC [18,19]. There are also extra-intestinal infections such as bacteraemia and urinary tract infection due to *E. albertii* [20,21]. Some studies have shown that *E. albertii* is possibly a waterborne and/or foodborne enteric pathogen since it was isolated from contaminated water [17], pork, duck meat, mutton and chicken meat [7,22].

*E. albertii* may carry several virulence-associated genes, such as *eae*, encoding an outer membrane protein, intimin that binds to the intestinal epithelium; cytolethal distending toxin, which is associated with persistent colonization, invasion and disease severity; and genes encoding two different type III secretion systems (T3SSs) [17,23]. *E. albertii* also produces Shiga toxin 2 (a or f variety), suggesting that this pathogen has the potential to cause severe diseases such as haemolytic uremic syndrome and haemorrhagic colitis in humans, similar to Shiga toxin-producing *E. coli* (STEC) [24].

In this study, we report the detection and characterization of *E. albertii* isolates from hospitalized paediatric diarrhoeal cases in Kolkata, India. We performed whole-genome sequence (WGS) analysis and made a robust intraspecies genomic comparison. These data provide insights into the genomic variation, virulence and antimicrobial resistance mechanisms of *E. albertii* strains.

## Methods

### Clinical stool specimens

Stool specimens were collected from acute diarrhoeal patients hospitalized at the B. C. Roy Children Hospital, Kolkata, India. Diarrhoeal patients typically had passage of >3 loose or liquid stools per day with no, some or severe dehydration as defined by the World Health Organization guidelines [25]. Faecal specimens were collected using sterile catheters in McCartney bottles or rectal swabs were collected in Cary Blair medium (HiMedia, India) and processed in the laboratory within 2 h for common enteric pathogens.

We have used conventional bacterial culture methods followed by biochemical or serological characterization of the pathogens. In brief, stool specimens and the enriched cultures were plated on the xylose lysine desoxycholate/Hekton enteric, thiosulphate citrate bile salts sucrose, *Aeromonas* (Ryan), Campy-BAP agar media for the isolation of *Salmonella/Shigella* spp., vibrios, *Aeromonas* spp. and campylobacters, respectively. Colonies grown on MacConkey agar were tested in the PCR assay for the detection of enterotoxigenic *E. coli* (ETEC), EPEC, EHEC, enteroinvasive *E. coli* (EIEC) and enteroaggregative *E. coli* (EAEC). Enteric viruses were detected by ELISA and multiplex reverse transcriptase PCR assay as described before [26].

### Isolation and identification of *E. albertii*

MacConkey agar was used for detection of common enteric pathogens and *E. albertii*. Faecal specimens were inoculated on MacConkey agar (Difco, USA) and incubated at 37 °C for 16–18 h. If available, three typical non-lactose fermenting colonies per sample from the MacConkey agar plate were picked and subcultured on Luria Bertani agar (LBA; Difco, USA). Colonies from LBA were tested for biochemical properties by conventional methods [27] using triple sugar iron (TSI) slants, motility and ornithine decarboxylase, lysine decarboxylase, Simmons citrate, urea and tryptophan broth for indole production. Additional tests were carried out using the Vitek-2 compact system (bioMérieux, Marcy l’Etoile, France). For PCR assay, colonies grown on LBA plates were suspended in 500 µl of sterile distilled water or PBS (pH 7.2) in 1.5 ml microfuge tubes. The bacterial suspension was boiled in a water bath for 10 min and then snap-chilled for 5 min. The bacterial suspension was centrifuged at 8 000 r.p.m. for 10 min. The supernatant was used as the DNA template. The DNA template was used in a multiplex PCR assay for the detection of virulence marker genes, such as CVD432 and *aaiC* (for EAEC), *eae* and *bfpA* (for EPEC) and *elt* and *est* (for ETEC) [28]. Simplex PCR assay was performed for *stx1* and *stx2* (for EHEC), *ipaH* (for EIEC) and cytolethal distending toxin (*cdt*) gene (for *E. albertii*) [29].

### Antimicrobial susceptibility testing

Antimicrobial susceptibility testing (AST) was performed in accordance with the Clinical and Laboratory Standards Institute [30] by disc diffusion method using commercially available discs (Becton Dickinson Company, USA), namely ampicillin (AMP), ceftriaxone (CRO), cefotaxime (CTX), ceftazidime (CAZ), chloramphenicol (CHL), nalidixic acid (NA), ciprofloxacin (CIP), ofloxacin (OFX), norfloxacin (NOR), meropenem (MEM), streptomycin (STR), erythromycin (E), azithromycin (AZM), gentamycin (GM), tetracycline (TET), doxycycline (D) and trimethoprim/sulfamethoxazole (SXT). *E. coli* ATCC 25922 was used as a control in the AST.

### O-serogrouping and *in silico* serotyping of *E. coli* isolates

To check for possible somatic antigen cross reactivity with *E. coli*, serogrouping was done using *E. coli* O-serogrouping kit by slide agglutination test (Denka-Seiken Co., Ltd., Tokyo, Japan). The kit consists of 8 O-polyvalent and 43 monospecific antisera. For *in silico* serotyping, we used the WGS and analysed the data with ECTyper version 1.0.0 with database version 1.0 [31]. The default settings were used for the serotyping.

### Pulsed-field gel electrophoresis (PFGE)

PFGE was performed using a CHEF-Mapper (Bio-Rad, USA) according to the Pulse-Net standardized protocol [32]. PFGE was made by *Xba*I-digested genomic DNA of *E. albertii* isolates and the *E. albertii* ATCC 19982 strain. PFGE images were saved by using a Gel Doc XR system (Bio-Rad). The PFGE gel images were analysed using the BioNumerics software version 5.0 (Applied Maths, Sint-Martens-Latem, Belgium) by normalizing and aligning the peaks of the *Salmonella enterica* serovar Braenderup H9812 size standard. The dice-coefficient method was used to check the banding similarity of the isolates and unweighted pair group method with arithmetic mean was used to calculate the clustering correlation coefficients between the isolates. The banding pattern of PFGE has been defined as follows: identity=level of similarity is 100%, near identity=level of similarity is 80-99% and similarity=level of similarity is 60-79%.

### Genomic sequencing, assembly and annotation

Genomic DNA libraries were prepared using the Lotus DNA Library Prep Kit (Integrated DNA Technologies, Coralville, IA, USA) and NEBNext Multiplex Oligos for Illumina (96 Unique Dual Index Primer Pairs) (New England BioLabs Japan, Tokyo, Japan). The libraries were sequenced on an Illumina HiseqX Ten platform (Illumina, San Diego, CA, USA) to generate 151 bp paired-end reads. Genome assembly was performed using the Platanus_b 1. 3. 2 [33]. We also included 635 and 2 *E. albertii* assemblies from the Enterobase and the National Center for Biotechnology Information (NCBI) databases, respectively. Genomes were removed due to low quality as judged by CheckM v1. 2. 0 [34] with a cutoff of less than 97% completeness or more than 5% contamination. We also removed two genomes in which the whole-genome average nucleotide identity against the type strain of *E. albertii* (strain NBRC107761) computed using FastANI version 1.33 [35] was less than 97%. In the analysis, 637 genomes were annotated using Prokka 1. 14. 6 [36].

### Multilocus sequence typing (MLST)

To determine the MLST, we utilized SRST2 version 0.2.0 with Escherichia_coli#1.fasta as the reference database [37]. The minimum coverage threshold was set at 98%, and the maximum deviation allowed was less than 2%.

### Phylogenetic analysis

To construct a core gene-based phylogenetic tree, pan-genomic analysis was performed using Roary 3. 13. 3 with a 90% sequence identity threshold. Single nucleotide polymorphism (SNP) sites were extracted from the core gene alignment using snp-sites [38], and a maximum likelihood (ML) phylogenetic tree was constructed using RAxML-NG v. 1. 1 [39]. RhierBAPS 1.1.3 was used to analyse population structure [40]. The ML phylogenetic tree was displayed and annotated using iTOL v6.6 [41].

### Identifications of virulence and antimicrobial resistance genes and plasmid typing

Presence of T3SS effector genes and the other *E. coli* virulence genes was analysed by tblastn 2.12.0+ and blastn 2.12.0+ searches, respectively, with a threshold of 80% identity and 60% coverage using in-house databases described previously [42]. Antimicrobial resistance genes (ARGs) and plasmid types were identified by ABRicate v1.0.1 (https://github.com/tseemann/abricate) using the ARG-ANNOT database [43] and PlasmidFinder database [44] with default parameters. Quinolone-resistant mutants in the quinolone-resistance determining region (QRDR) of *gyrA* and *parC* genes were analysed using AMRFinderPlus 3.11.14 with default settings [45].

### Statistical analysis

The difference between proportions was assessed by entering the values in a 2×2 contingency table and applying Chi-square test or Fisher’s exact test as appropriate. A *P* value of≤0.05 was considered significant.

## Results

### Clinical characterization of *E. albertii* isolates

Age, gender and clinical presentation of the patients, like stool characteristics, dehydration status, fever and abdominal pain, are recorded in Table 1. A total of 854 rectal swabs/stool samples were collected from children<5 years of age with acute diarrhoea during June 2021–June 2022. A total of ten (1.2%) *E. albertii* isolates were identified. *E. albertii* isolation was slightly higher during the period between summer and monsoon (March–August) (*n*=7/854; 0.81%) months as compared to autumn months (September–November) (*n*=3/854; 0.35%) (*P*=0.34). Children aged 1–12 months (*n*=8/854; 0.94%) showed a slightly higher isolation rate of *E. albertii* than children aged 12–60 months (*n*=2/854; 0.23%) (*P*=0.11). Male children (*n*=7/854; 0.81%) were affected slightly more than the female children (*n*=3/854; 0.35%) (*P*=0.34). The most common type of diarrhoea observed in patients with *E. albertii* infection was watery diarrhoea (*n*=6/10, 60%), followed by diarrhoea containing mucus (30%) and diarrhoea with blood and mucus (10%). All the patients with *E. albertii* infections had moderate dehydration (100%). About 20% of the patients had abdominal pain and fever (Table 1). Most of the patients given fluoroquinolone, metronidazole and probiotics responded to the treatment. All the patients recovered and were discharged in a stable condition.

**Table 1.** Clinical features of paediatric diarrhoeal patients infected with *E. albertii*

Strain ID	Date of isolation	Age	Gender	Clinical feature				Dehydration status		Fever		Abdominal pain		Treatment
				Watery	Loose	Bloody	Mucoid	Severe	Some	Yes	No	Yes	No	
BCH12731	05.06.2021	11 M	M	+	−	−	−	−	+	+	−	+	−	Racecadotril+*Saccharomyces boulardii*, zinc, probiotics
BCH12846	12.07.2021	2 Y	M	+	−	−	−	−	+	−	+	−	+	Ondansetron+zinc, probiotics
BCH12925	23.08.2021	8 M	F	−	−	−	+	−	+	−	+	−	+	Norfloxacin+metronidazole+ zinc, probiotics
BCH13002	29.09.2021	10 M	M	−	−	+	−	−	+	−	+	−	+	Sulfamethoxazole+trimethoprim+ zinc, probiotics
BCH13327	31.03.2022	8 M	M	+	−	−	−	−	+	−	+	−	+	Cefpodoxime proxetil+lansoprazole+ zinc, probiotics
BCH13052	02.11.2021	10 M	M	+	−	−	−	−	+	−	+	−	+	Levofloxacin+lansoprazole+ zinc, probiotics
BCH13279	15.03.2022	9 M	M	+	−	−	−	−	+	+	−	+	−	Norfloxacin+metronidazole+ondansetron+zinc,probiotics
BCH13029	20.10.2021	5 Y	M	−	−	−	+	−	+	−	+	−	+	norfloxacin+metronidazole+zinc, probiotics
BCH13559	15.06.2022	8 M	F	−	−	−	+	−	+	−	+	−	+	Ondansetron+lansoprazole, zinc, probiotics
BCH13564	17.06.2022	9 M	F	+	−	−	−	−	+	−	+	−	+	Racecadotril+*Saccharomyces boulardii*, zinc, probiotics

### Biochemical properties and PCR of *E. albertii* isolates

Biochemical properties of the ten *E. albertii* isolates are listed in Table 2. All *E. albertii* isolates were non-motile and positive for lysine decarboxylase, ornithine decarboxylase and indole while being negative for citrate, H_2_S and urease. Further biochemical analysis of the ten *E. albertii* isolates was made using the Vitek-2 compact system Gram-negative card. This system is not programmed for the detection of *E. albertii*, and there were no consistent phenotypic differences found between *E. albertii* (isolates and controls) and *Escherichia fergusonii*, *E. coli* and *Shigella* controls. However, all *E. albertii* isolates were negative for the following Vitek-2 tests: alpha-glucosidase (AGLU), glutamyl arylamidase pNA (AGLTp), d-tagatose (dTAG), lipase (LIP), Ala-Phe-Pro-acrylamidase (APPA), urease (URE), l-arabadose (IARL), palatinose (PLE), citrate sodium (CIT), 5-keto-d-gluconate (5 KG), H_2_S production, beta-*N*-acetyl-glucosaminidase (BNAG), beta-*N*-acetyl-galactosaminidase (NAGA), beta-glucosidase (BGLU), beta-xylosidase (BXYL), beta-alanine arylamidase pNA (BAIap), sucrose/sucrose (SAC), malonate (MNT), l-histidine assimilation (IHISa) and Glu-Gly-Arg-arylamidase (GGAA).

**Table 2.** Biochemical properties of *E. albertii* isolated from paediatric diarrhoeal patients

Strain ID	Organism	TSI	Motility	Ornithine decarboxylase	Lysine decarboxylase	Simmons citrate	Urease	Indole	*E. albertii* cytolethal distending toxin (*Ea-cdt*) gene	EPEC intimin (*eae*) gene
BCH12731	*E. albertii*	K/A, G^+^, H_2_S^-^	−	+	+	−	−	+	+	+
BCH12846	*E. albertii*	K/A, G^+^, H_2_S^-^	−	+	+	−	−	+	+	+
BCH12925	*E. albertii*	K/A, G^-^, H_2_S^-^	−	+	+	−	−	+	+	+
BCH13002	*E. albertii*	K/A, G^+^, H_2_S^-^	−	+	+	−	−	+	+	+
BCH13327	*E. albertii*	K/A, G^+^, H_2_S^-^	−	+	+	−	−	+	+	+
BCH13052	*E. albertii*	K/A, G^+^, H_2_S^-^	−	+	+	−	−	+	+	+
BCH13279	*E. albertii*	K/A, G^-^, H_2_S^-^	−	+	+	−	−	+	+	+
BCH13029	*E. albertii*	K/A, G^-^, H_2_S^-^	−	+	+	−	−	+	+	+
BCH13559	*E. albertii*	K/A, G^+^, H_2_S^-^	−	+	+	−	−	+	+	+
BCH13564	*E. albertii*	K/A, G^+^, H_2_S^-^	−	+	+	−	−	+	+	+
ATCC 19982	*E. albertii*	K/A, G^+^, H_2_S^-^	−	+	+	−	−	+	+	+
48–23	*E*. *fergusonii*	K/A or A/A, G^+^, H_2_S^-^	+	+	+	−	−	+	−	−
PV14-161	*E. coli*-clade 1	K/A or A/A, G^+^, H_2_S^-^	+	+	+	−	−	+	−	−
BCH 8159	EIEC/*Shigella* spp.	K/A, G^-^, H_2_S^-^	−	−	−	−	−	−	−	−

EIEC: enteroinvasive *E*. *coli*; K: alkaline slant; A: acid butt; G: gas; H_2_S: hydrogen sulphide; EPEC: enteropathogenic *E. coli*; ‘+’: positive; ‘−’: negative.

All ten isolates were positive for the species-specific cytolethal distending toxin gene (*cdt*) and the intimin-encoding gene (*eae*). None of the isolates were positive for the other virulence-encoding genes specific for diarrhoeagenic *E. coli* and *Shigella* (*stx1*, *stx2*, *bfpA*, *ipaH*, *elt*, *est*, *aaiC* or *CVD432*) (Table 2).

### Antimicrobial resistance profile and PFGE analysis of *E. albertii*

The antimicrobial resistance and MLST profiles of *E. albertii* isolates are shown in Table 3. The AST of *E. albertii* showed that most of the isolates were susceptible or reduced susceptible to all the antibiotics, except for erythromycin (80%), tetracycline (50%), nalidixic acid (40%), ampicillin (40%), doxycycline (30%), ceftriaxone (20%), azithromycin (10%) and trimethoprim/sulfamethoxazole (10%). Agglutination test showed only two isolates belonged to *E. coli* serogroup O115, and others were untypeable. Of the ten *E. albertii* strains, six (60%) were identified as the sole pathogen and the remaining isolates were mixed with other pathogens such as EAEC, adenovirus and rotavirus.

**Table 3.** The antimicrobial resistance profile and MLST profile of *E. albertii* isolated from paediatric diarrhoeal patients

Strain ID	Agglutination with *E. coli* ‘O’ serogroup antisera	MLST	AMR profile	Reduced susceptibilityprofile	Infection statusSole/Mixed
BCH12731	ONT	4619	NA, TET, AM, CRO, E	CIP, NOR, OFX, S, AZM, D, CTX	Mixed (Adenovirus, EAEC)
BCH12846	ONT	4619	NA, E	S	Mixed (Adenovirus)
BCH12925	ONT	4596	NA, CIP, TET, AM, CRO, E	NOR, OFX, S, D, CTX	Sole
BCH13002	ONT	12292	NA, E	S, AZM	Sole
BCH13327	ONT	NF	–	S, E	Sole
BCH13052	115	1846	E	S	Sole
BCH13279	ONT	4596	TET, D	S, E	Mixed (Rotavirus)
BCH13029	ONT	1996	E, AZM	TET, S, D	Sole
BCH13559	ONT	4596	TET, AM, SXT, E, D	NA, S, AZM	Mixed (EAEC)
BCH13564	115	1846	TET, AM, E, D	S	Sole

AM, ampicillin; AZM, azithromycin; CIP, ciprofloxacin; CRO, ceftriaxone; CTX, cefotaxime; D, doxycycline; E, erythromycin; EAECenteroaggregative *Escherichia coli*NA, nalidixic acid; NFnot foundNOR, norfloxacin; OFX, ofloxacin; ONTuntypableS, streptomycin; SXT, trimethoprim–sulfamethoxazole; TET, tetracycline

**Fig. 1.**
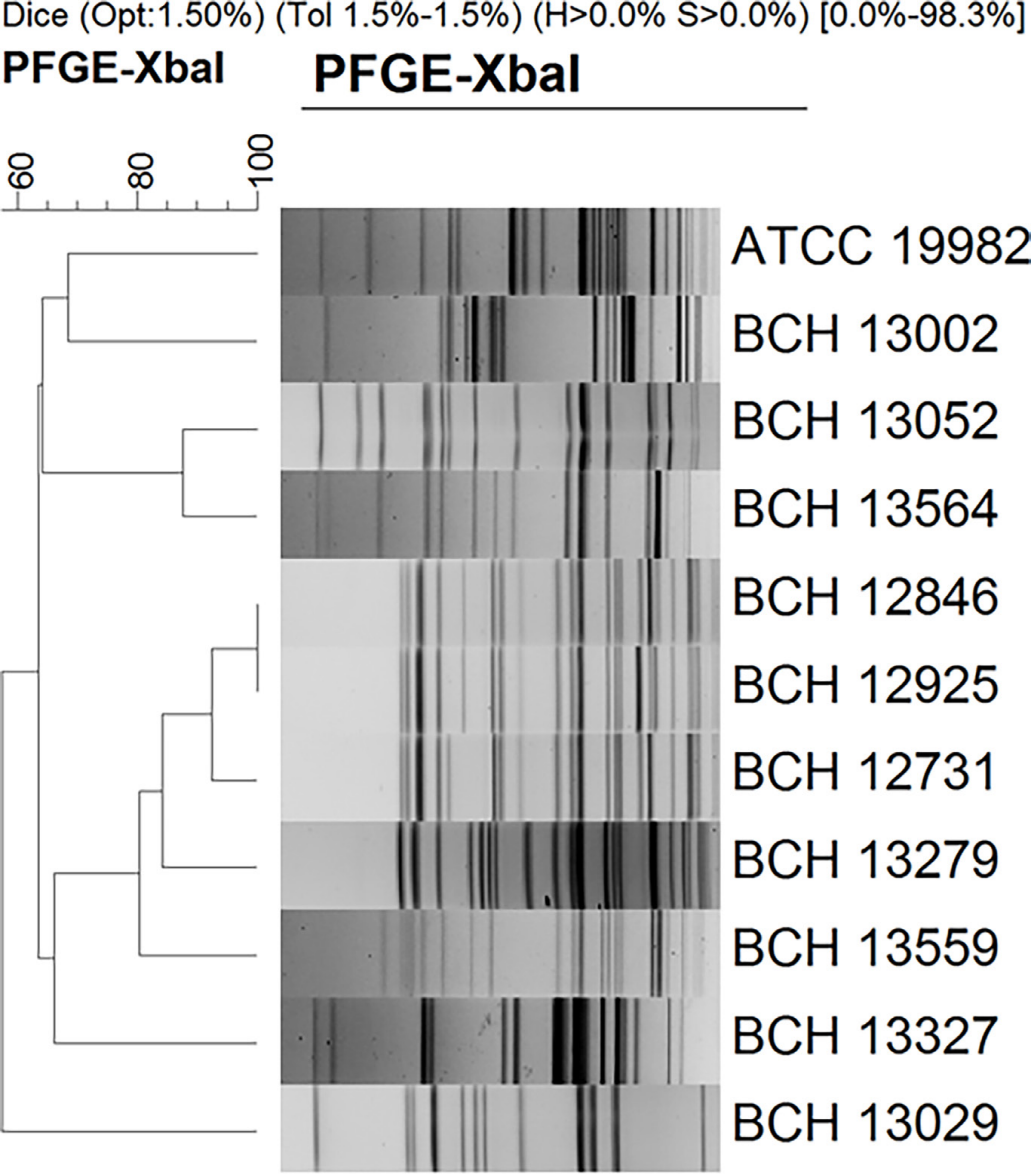
PFGE analysis of *Xba*I-digested genomic DNA of *E. albertii* isolates from diarrhoeal faecal samples of paediatric patients in Kolkata, India.

The PFGE profiles of *E. albertii* strains showed distinct patterns. When tested using dice-coefficient methods, ~60% similarity value was obtained with *E. albertii* isolates (Fig. 1). There was no profile match among the isolates except for two isolates that showed identity and belonged to the ONT serogroup (isolates BCH 12846 and BCH 12925), and nearly identical PFGE profiles were obtained for another two isolates belonging to the O115 serogroup (isolates BCH 13052 and BCH 13564).

### Whole genome sequence (WGS) and MLST of the *E. albertii*

The draft genome sequences of the ten *E. albertii* isolates were determined. Details of WGS information are provided in Table 4. The genome sizes ranged from 4 730 kb to 5 358 kb. Consistent with the antisera-based serotyping, *in silico* serotyping identified two isolates as O115 and the others remained untypeable by both methods (Table 3). In the MLST analysis, nine isolates were grouped into five different sequence types (STs): ST4596 (*n*=3), ST1846 (*n*=2), ST4619 (*n*=2), ST1996 (*n*=1) and ST12292 (*n*=1), and the remaining isolates were not grouped into any of the known STs (Table 3).

**Table 4.** Whole-genome sequencing information for *E. albertii*

Characteristics		*E. albertii* isolate								
	BCH12731	BCH12846	BCH13029	BCH13002	BCH13279	BCH13559	BCH12925	BCH13052	BCH13564	BCH13327
Sequence size (bp)	4 730 027	4 730 044	4 731 438	4 872 076	4 792 401	4 876 092	4 997 797	5 094 325	5 358 185	4 912 038
No. of scaffolds	94	94	95	135	219	219	269	224	363	86
N50 (bp)	261 073	366 116	217 629	228 167	151 503	162 653	148 212	132 613	120 841	532 878
G+C content (mol%)	49.8	49.8	49.8	49.7	49.5	49.5	49.3	49.7	49.8	49.6
Longest scaffold size (bp)	630 935	630 935	453 892	523 098	301 566	383 521	405 928	365 599	384 372	904 939
Number of CDSs	4456	4453	4405	4553	4409	4498	4619	4749	4971	4574
Number of rRNA	16	16	6	8	10	12	10	10	9	12
Number of tRNA	91	95	87	100	87	95	93	99	97	81

### Virulence genes identified in *E. albertii* isolates

To evaluate the potential virulence of the *E. albertii* isolates, we examined the presence of known *E. coli* virulence genes (Table 5). All isolates were identified for *eaeA*, *cdt-IIA* and *paa* genes. Genes for the seven known locus of enterocyte effacement (LEE) encoded effectors and a positive regulator of LEE gene expression (*pchA*) were also identified in all the isolates. In addition, various combinations of non-LEE effectors were present in the *E. albertii* isolates: *cif* (*n*=1), *espH* (*n*=7), *espJ* (*n*=3), *espK* (*n*=3), *espM2* (*n*=6), *espV* (*n*=4), *espW* (*n*=2), *nleA* (*n*=8), *nleB1* (*n*=3), *nleB2* (*n*=4), *nleC* (*n*=5), *nleD* (*n*=5), *nleE* (*n*=3), *nleF* (*n*=1), *nleG3* (*n*=2), *nleG8* (*n*=6), *nleH1* (*n*=6), *nleH2* (*n*=1) and *tccP* (*n*=2). In addition, virulence genes known to be present in the EHEC virulence plasmids [46], such as *efa1* (adhesin, *n*=4), *espC* (serine protease, *n*=4) and *lpxR* (lipid A 3′-O-deacylation, *n*=2), were also detected in several isolates.

**Table 5.** Virulence gene profiles of *E. albertii* isolated from paediatric diarrhoeal patients

Gene		BCH12731	BCH12846	BCH13029	BCH13002	BCH13279	BCH13559	BCH12925	BCH13052	BCH13564	BCH13327
**Intimin**	***eae***	**+**	**+**	**+**	**+**	**+**	**+**	**+**	**+**	**+**	**+**
**Non-LEE effectors**	***cif***	−	−	−	−	−	−	−	−	−	**+**
	***espH***	**+**	**+**	**+**	**+**	**+**	**+**	**+**	−	−	−
	***espJ***	−	−	−	−	**+**	**+**	**+**	−	−	−
	***espK***	**+**	**+**	−	**+**	−	−	−	−	−	−
	***espM2***	**+**	**+**	−	**+**	−	−	−	**+**	**+**	**+**
	***espV***	**+**	**+**	**+**	**+**	−	−	−	−	−	−
	***espW***	−	−	−	−	−	−	−	**+**	**+**	−
	***nleA***	**+**	**+**	**+**	−	**+**	**+**	**+**	**+**	**+**	−
	***nleB1***	−	−	−	−	−	−	−	**+**	**+**	**+**
	***nleB2***	**+**	**+**	**+**	**+**	−	−	−	−	−	−
	***nleC***	−	−	−	−	**+**	**+**	**+**	**+**	**+**	−
	***nleD***	−	−	**+**	**+**	**+**	**+**	**+**	−	−	−
	***nleE***	−	−	−	−	−	−	−	**+**	**+**	**+**
	***nleF***	−	−	−	**+**	−	−	−	−	−	−
	***nleG3***	−	−	−	−	−	−	−	**+**	**+**	−
	***nleG8***	**+**	**+**	−	**+**	−	−	−	**+**	**+**	**+**
	***nleH1***	−	−	**+**	**+**	**+**	**+**	**+**	−	−	**+**
	***nleH2***	−	−	−	**+**	−	−	−	−	−	−
	***tccP***	−	−	−	−	**+**	**+**	−	−	−	−
**Regulator**	***pchA***	**+**	**+**	**+**	**+**	**+**	**+**	**+**	**+**	**+**	**+**
**Plasmid adhesin**	***efa1***	**+**	**+**	**+**	**+**	−	−	−	−	−	−
**Enterotoxin**	***espC***	**+**	**+**	**+**	**+**	−	−	−	−	−	−
**Lipid A 3′-O-deacylase**	***lpxR***	−	−	−	−	−	−	−	**+**	**+**	−
**Adhesin**	***paa***	**+**	**+**	**+**	**+**	**+**	**+**	**+**	**+**	**+**	**+**
**Cytolethal distending toxin**	***cdt-IIA***	**+**	**+**	**+**	**+**	**+**	**+**	**+**	**+**	**+**	**+**

### ARGs in *E. albertii* isolates

In the WGS analysis, it was observed that seven isolates tested positive for the presence of ARGs genes that encode resistance to beta-lactamases (*bla*_CTX-M-55_, *n*=1; *bla*_TEM-105_, *n*=2), quinolones (*qnrS1*, *n*=3; *qnrB32*, *n*=1), sulphonamides (*sul2*, *n*=2), trimethoprim (*dfrA1*, *n*=1) and tetracyclines (*tetA*, *n*=4) (Table 6). In addition, four isolates were found to have point mutations in the *gyrA* of the QRDR within the subunits constituting topoisomerases II (Table 6). Five multidrug-resistant isolates carried ARGs conferring resistance to different classes of antimicrobials.

**Table 6.** ARG profiles of *E. albertii* isolated from paediatric diarrhoeal patients

Antimicrobial class	Gene	BCH12731	BCH12846	BCH13029	BCH13002	BCH13279	BCH13559	BCH12925	BCH13052	BCH13564	BCH13327
**Aminoglycosides**	***aadA1***	−	−	−	−	−	**+**	−	−	−	−
**Beta-lactamases**	***bla*_CTX-M-55_**	−	−	−	−	−	−	**+**	−	−	−
	***bla*_TEM-105_**	−	−	−	−	−	**+**	−	−	**+**	−
**Fluoroquinolones**	***qnrS1***	−	−	−	−	**+**	−	**+**	−	**+**	−
	***qnrB32***	−	−	−	−	−	−	**+**	−	−	−
**Sulphonamides**	***sul2***	−	−	−	−	−	**+**	**+**	−	−	−
**Tetracyclines**	***tetA***	−	−	−	−	**+**	**+**	**+**	−	**+**	−
	***tetR***	−	−	−	−	**+**	**+**	**+**	−	**+**	−
**Trimethoprim**	***dfrA1***	−	−	−	−	−	**+**	−	−	−	−

Table 7 shows the results of conventional AST and WGS-based detection of AMR. Even though the AST showed resistance to macrolide, beta-lactamases and tetracycline in some of the *E. albertii* isolates, the respective ARGs could not be detected in the WGS analysis. Conversely, two isolates carrying ARGs that encode resistance to aminoglycosides and beta-lactamase did not exhibit resistance in the AST similar to the findings of point mutations in the *gyrA* gene, which were associated with resistance to quinolone class of antibiotics.

**Table 7.** AST and WGS-based ARG detection in *E. albertii* from paediatric diarrhoeal patients

Antimicrobial	*E. albertii* isolate and resistance																			
	BCH12731		BCH12846		BCH12925		BCH13002		BCH13029		BCH13052		BCH13279		BCH13327		BCH13559		BCH13564	
	AST	WGS	AST	WGS	AST	WGS	AST	WGS	AST	WGS	AST	WGS	AST	WGS	AST	WGS	AST	WGS	AST	WGS
Aminoglycosides	–	–	–	–	–	–	–	–	–	–	–	–	–	–	–	–	–	+	–	–
Beta-lactamases should be beta-lactam	+	–	–	–	+	+	–	–	–	–	–	–	–	–	–	–	–	+	–	+
Colistin	–	–	–	–	–	–	–	–	–	–	–	–	–	–	–	–	–	–	–	–
Fosfomycin	–	–	–	–	–	–	–	–	–	–	–	–	–	–	–	–	–	–	–	–
Quinolones	+	+	+	+	+	+	+	+	–	–	–	+	–	–	–	–	–	–	–	+
Macrolides	+	–	+	–	+	–	+	–	–	–	+	–	–	–	+	–	+	–	+	–
Phenicols	–	–	–	–	–	–	–	–	–	–	–	–	–	–	–	–	–	–	–	–
Rifampicin	–	–	–	–	–	–	–	–	–	–	–	–	–	–	–	–	–	–	–	–
Sulphonamides	–	–	–	–	–	+	–	–	–	–	–	–	–	–	–	–	+	+	–	–
Trimethoprim	–	–	–	–	–	–	–	–	–	–	–	–	–	–	–	–	+	+	–	–
Tetracyclines	+	–	–	–	+	+	–	–	–	–	–	–	+	+	–	–	+	+	+	+

### Plasmid typing in *E. albertii* isolates

The plasmid types identified in the draft genome obtained in this study include Col(MG828) (*n*=1), ColRNAI (*n*=6), IncB/O/K/Z_4 (*n*=1), IncFIA_1 (*n*=3), IncFIB(AP001918) (*n*=3), IncFIB(pB171) (*n*=3), IncFII_1 (*n*=2), IncFII_1_pSFO (*n*=2), IncFII(29)_1_pUTI89 (*n*=2), IncFII(pHN7A8) (*n*=3), IncFII(pSE11) (*n*=3), IncI_Gamma_1 (*n*=1), IncI1_1_Alpha (*n*=2), IncN_1 (*n*=1), IncX1_4 (*n*=1) and IncY (*n*=2) from seven isolates (Table 8). Among these, the isolate BCH12925 contained the most diverse set of plasmids, with ten identified plasmid types. In contrast, no plasmid types were detected in isolates BCH12731, BCH12846 and BCH13002 in cluster 1.

**Table 8.** Plasmid replicon type of *E. albertii* strains isolated from paediatric diarrhoeal patients

Plasmid replicon type	BCH12731	BCH12846	BCH12925	BCH13002	BCH13029	BCH13052	BCH13279	BCH13327	BCH13559	BCH13564
**Col(BS512**)	−	−	−	−	−	−	−	−	−	−
**Col(KPHS6**)	−	−	−	−	−	−	−	−	−	−
**Col(MG828**)	−	−	+	−	−	−	−	−	−	−
**Col(MP18**)	−	−	−	−	−	−	−	−	−	−
**Col156**	−	−	−	−	−	−	−	−	−	−
**Col440I**	−	−	−	−	−	−	−	−	−	−
**Col440II**	−	−	−	−	−	−	−	−	−	−
**Col8282**	−	−	−	−	−	−	−	−	−	−
**ColE10**	−	−	−	−	−	−	−	−	−	−
**ColpVC**	−	−	−	−	−	−	−	−	−	−
**ColRNAI**	−	−	+	−	−	+	+	+	+	+
**IncB/O/K/Z_4**	−	−	−	−	−	−	−	−	−	+
**IncFIA_1**	−	−	+	−	−	−	+	−	+	−
**IncFIA(HI1)_1_HI1**	−	−	−	−	−	−	−	−	−	−
**IncFIB(AP001918)_1**	−	−	−	−	+	+	−	−	−	+
**IncFIB(K)_1_Kpn3**	−	−	−	−	−	−	−	−	−	−
**IncFIB(pB171)_1_pB171**	−	−	+	−	−	−	+	−	+	−
**IncFIB(pENTAS01)_1_pENTAS01**	−	−	−	−	−	−	−	−	−	−
**IncFIB(pHCM2)_1_pHCM2**	−	−	−	−	−	−	−	−	−	−
**IncFIC(FII)_1**	−	−	−	−	−	−	−	−	−	−
**IncFII_1**	−	−	−	−	+	−	−	+	−	−
**IncFII_1_pSFO**	−	−	−	−	−	+	−	−	−	+
**IncFII(29)_1_pUTI89**	−	−	+	−	−	−	−	−	+	−
**IncFII(pCoo)_1_pCoo**	−	−	−	−	−	−	−	−	−	−
**IncFII(pCRY)_1_pCRY**	−	−	−	−	−	−	−	−	−	−
**IncFII(pHN7A8)_1_pHN7A8**	−	−	+	−	−	+	−	+	−	−
**IncFII(pRSB107)_1_pRSB107**	−	−	−	−	−	−	−	−	−	−
**IncFII(pSE11)_1_pSE11**	−	−	+	−	−	−	+	−	+	−
**IncHI2_1**	−	−	−	−	−	−	−	−	−	−
**IncHI2A_1**	−	−	−	−	−	−	−	−	−	−
**IncI_Gamma_1**	−	−	−	−	−	−	−	−	+	−
**IncI1_1_Alpha**	−	−	−	−	−	−	+	−	−	+
**IncI2_1**	−	−	−	−	−	−	−	−	−	−
**IncI2_1_Delta**	−	−	−	−	−	−	−	−	−	−
**IncN_1**	−	−	+	−	−	−	−	−	−	−
**IncP1_3**	−	−	−	−	−	−	−	−	−	−
**IncQ1_1**	−	−	−	−	−	−	−	−	−	−
**IncR_1**	−	−	−	−	−	−	−	−	−	−
**IncX1_1**	−	−	−	−	−	−	−	−	−	−
**IncX1_4**	−	−	+	−	−	−	−	−	−	−
**IncX3_1**	−	−	−	−	−	−	−	−	−	−
**IncX4_1**	−	−	−	−	−	−	−	−	−	−
**IncX4_2**	−	−	−	−	−	−	−	−	−	−
**IncY_1**	−	−	+	−	−	−	−	−	+	−
**p0111_1**	−	−	−	−	−	−	−	−	−	−
**pEC4115_1**	−	−	−	−	−	−	−	−	−	−
**pENTAS02_1**	−	−	−	−	−	−	−	−	−	−
**RepA_1_pKPC-CAV1321**	−	−	−	−	−	−	−	−	−	−

### Comparative genomics of *E. albertii*

**Fig. 2.**
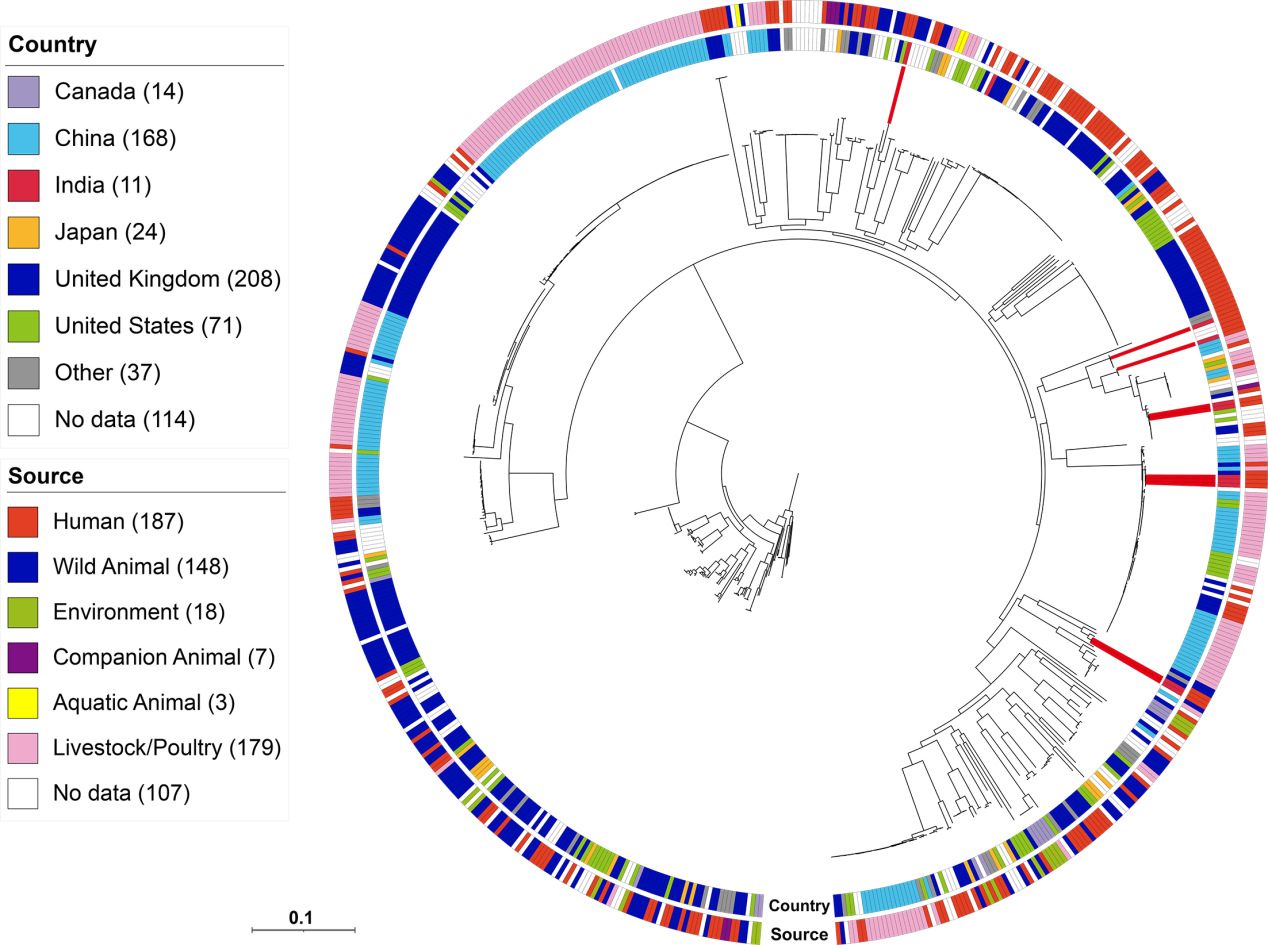
*E. albertii* lineages and their distribution in different geographic regions and source types are not clear. Red blocks represent the ten isolates obtained in this study.

**Fig. 3.**
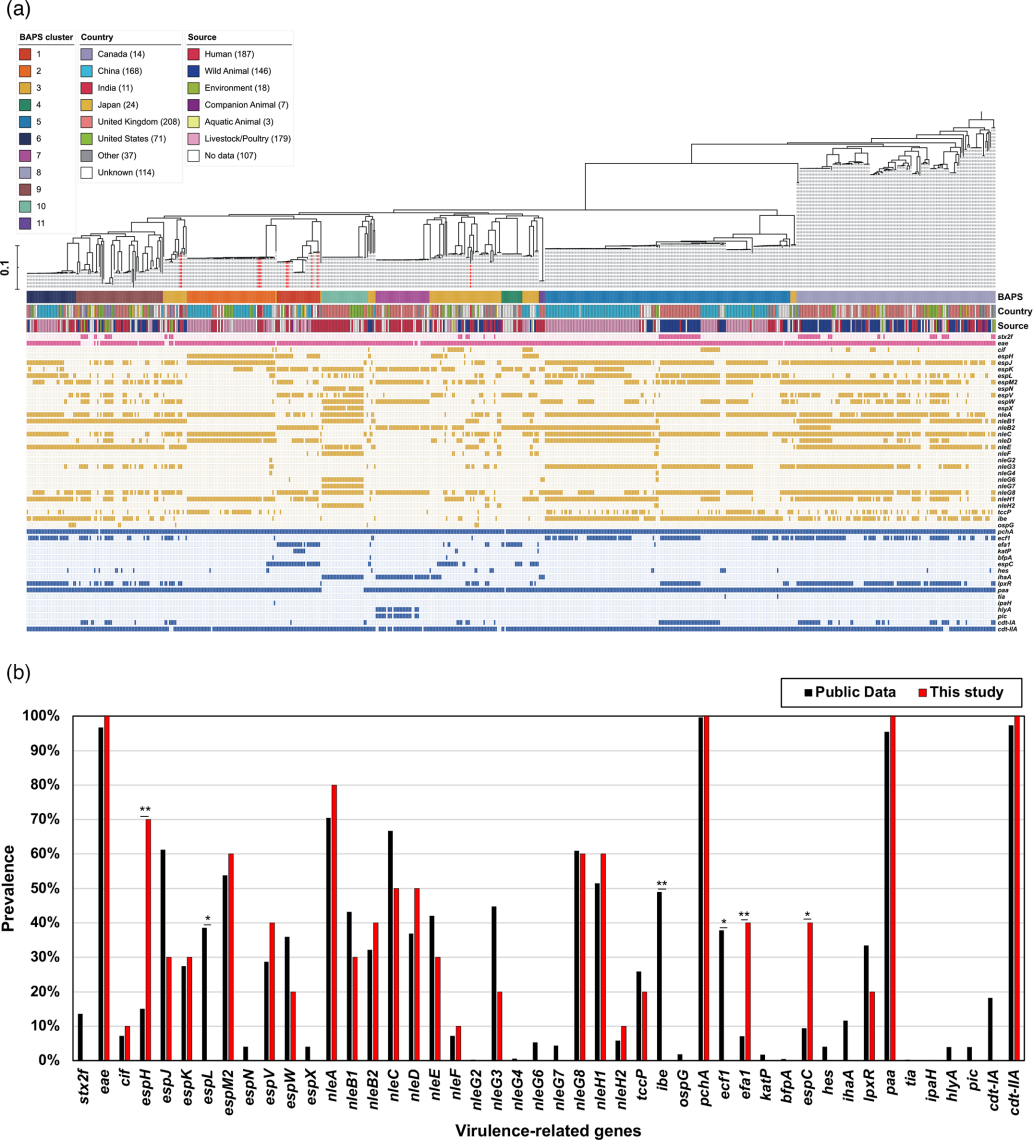
(**a**) Virulence genes and their distribution in *E. albertii* lineages isolated from diarrhoeal faecal samples of paediatric patients in Kolkata, India. Red lines indicate the ten isolates obtained in this study. (**b**) Comparison of the prevalence of each gene between the isolates in this study and those in public data. Asterisks indicate significant differences as determined by Fisher’s exact test (*P<0.05, **P<0.01).

**Fig. 4.**
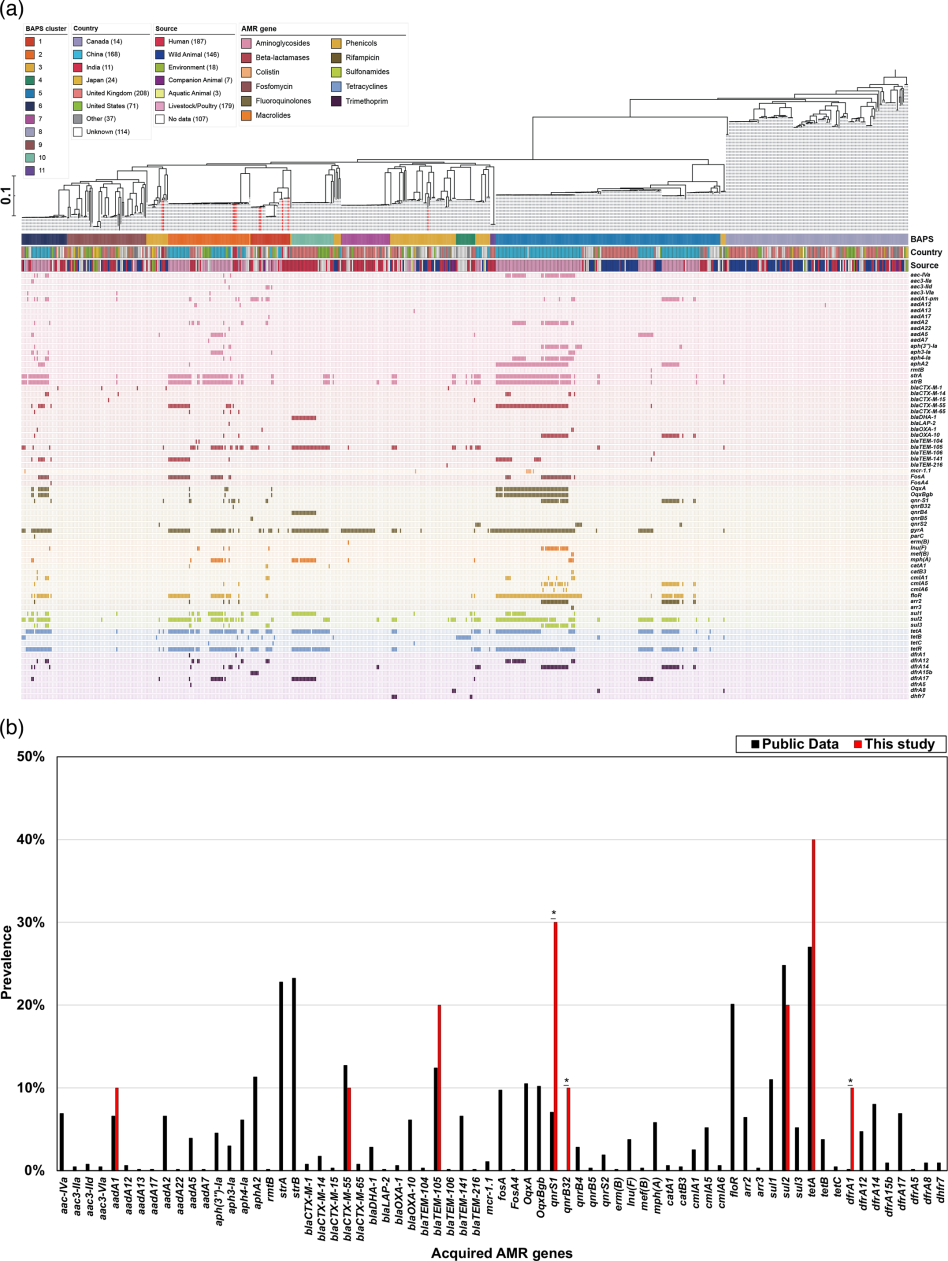
(**a**) Drug resistance genes and their prevalence in *E. albertii* isolated from diarrhoeal faecal samples of paediatric patients in Kolkata, India. The ten isolates from this study are shown as red lines. (**b**) Comparison of the prevalence of each gene between the isolates in this study and those in public data. Asterisks indicate significant differences as determined by Fisher’s exact test (*P<0.05, **P<0.01).

**Fig. 5.**
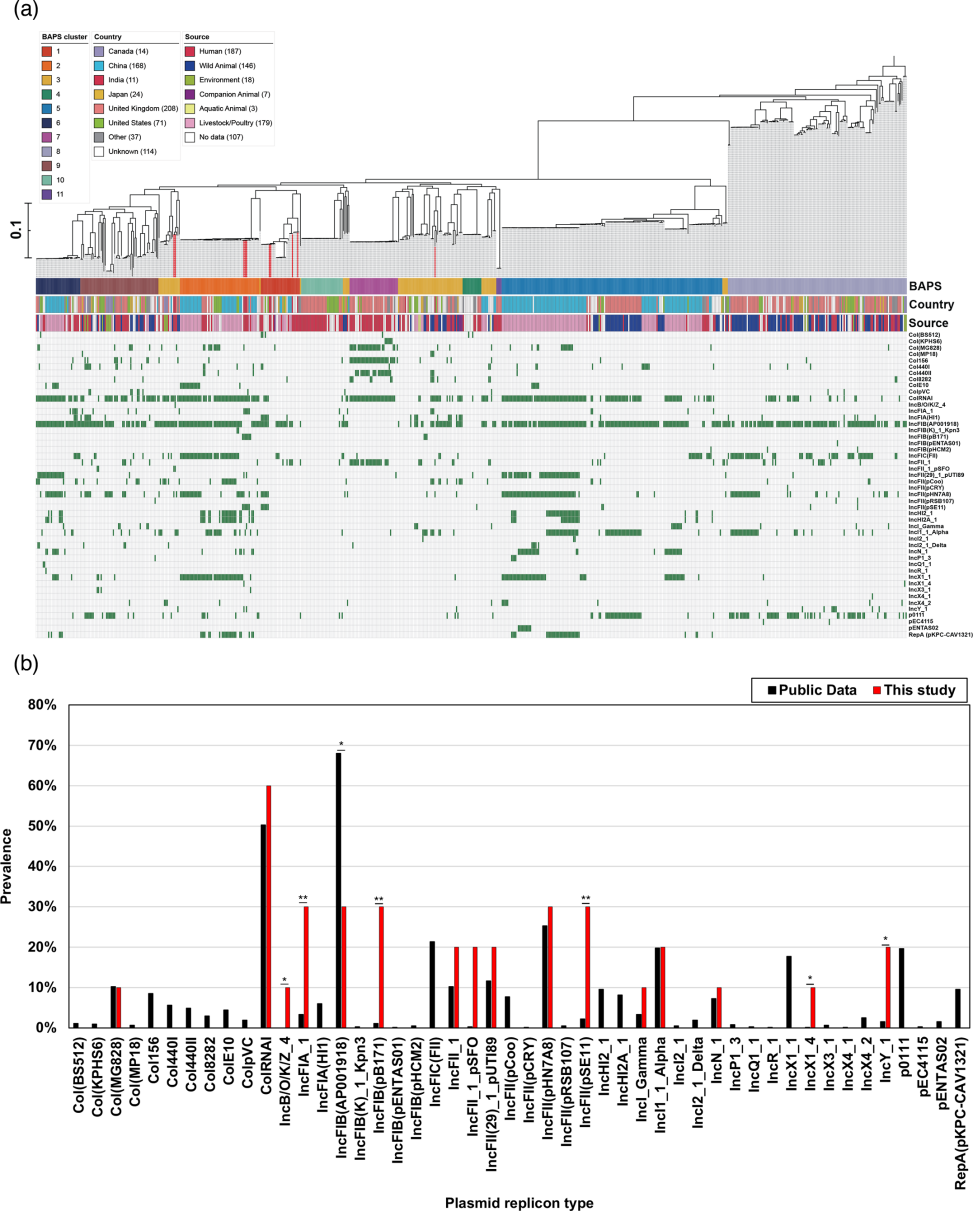
(**a**) The plasmid type and their prevalence in *E. albertii* isolates from diarrhoeal faecal samples of paediatric patients in Kolkata, India. The ten isolates from this study are shown as red lines. (**b**) Comparison of the prevalence of each gene between the isolates in this study and those in public data. Asterisks indicate significant differences as determined by Fisher’s exact test (*P<0.05, **P<0.01).

Comparative genomic analysis was made using WGS generated in this study and the available *E. albertii* genome sequences in the Enterobase/NCBI genome database. Of the 647 isolates analysed, 10 were from this study (Table S1). These isolates were from different countries from six different sources, including livestock/poultry (*n*=179; 28.1%), humans (*n*=177; 27.8%) and other sources. To analyse the population structure of *E. albertii*, an ML phylogenetic tree was constructed using SNP alignments spanning 70 388 bp (Fig. 2). Furthermore, all isolates, including those from the public database, were arranged in 11 clusters when tested using the Bayesian analysis of population structure (BAPS). Among these clusters, the isolates from this study were assigned to three specific clusters (BAPS cluster 1, *n*=4; cluster 2, *n*=3; cluster 3, *n*=3). Compared to the public data on the percentage of harbouring virulence genes (Table 5 and Fig. 3), the isolates in this study showed a significantly higher frequency of three virulence genes: *espC* (40% in this study vs. 9.4% in public data), *espH* (70% vs. 15.1%) and *efa1* (40% vs. 7.1%) (Fig. 3b). Four isolates belonging to BAPS cluster 1 harboured these genes, and only *espH* was also found in three isolates in cluster 2. In contrast, the proportion of three virulence genes was significantly lower in the isolates from this study compared to the public data: *espL* (0% in this study vs. 39% in public data), *ibe* (0% vs. 49%) and *ecf1* (0% vs. 38%) (Fig. 3b). Other differences were not significant for *espJ* (30% vs. 61.2%) and *nleG3* (20% vs. 45%), though these genes were also less prevalent in the isolates in this study than in the public data (Fig. 3b). Similarly, a comparison with public databases for ARGs (Table 6 and Fig. 4) showed that the *qnrS1* gene, which had a high percentage in the isolates of this study (30% vs. 7.1% in public data), was not disproportionately present in the cluster to which the isolates in this study belong to. The presence of ARGs (*strA*, *strB* and *floR*) was not uniform in the isolates placed in clusters 1 and 3. Interestingly, isolates in cluster 2 from this study did not possess these ARGs, despite high prevalence rates in other cluster 2 strains from public data: *strA* (77%, *n*=43/56), *strB* (79%, *n*=44/56), *floR* (55%, *n*=31/56) and *sul1* (34%, *n*=19/56). Conversely, *tetA* was common in the cluster 2 isolates from this study, as well as ARGs such as *qnrS1* (67%, *n*=2/3 vs. 11%, *n*=6/56 in public data), *qnrB32* (33%, *n*=1/3 vs. 0%, *n*=0/56) and *dfrA1* (33%, *n*=1/3 vs. 2%, *n*=1/56), which were infrequent in the public data for cluster 2. A comparison of plasmid type frequencies with public data revealed that several plasmid types had significantly higher percentages of isolates in this study: IncB/OKZ_4 (10% in this study vs. 0% in public data), IncFIA_1 (3.3% vs. 30%), IncFIB(pB171) (30% vs. 1.1%), IncFII(pSE11) (30% vs. 2.2%), IncX1_4 (10% vs. 0.2%) and IncY_1 (20% vs. 1.6%) (Table 8 and Fig. 5b). In contrast, the prevalence of IncFIB(AP001918) (30% vs. 68%) was significantly lower than in the public data (Table 8). Cluster-specific analysis showed that cluster 1 had overall low retention rates, cluster 2 exhibited high rates of plasmid type identification and cluster 3 showed moderate rates, indicating that the isolates in this study demonstrated a similar trend (Fig. 5a).

## Discussion

*E. albertii* is a recently defined member of the emerging *Enterobacteriaceae* and has been designated as one of the human diarrhoeal pathogens [1]. *E. albertii* was first isolated from a child with diarrhoea in Bangladesh, where it was initially identified as *H. alvei* by biochemical assays [4]. The *H. alvei*-like strains were subsequently reclassified as a new species, *E. albertii*, by DNA–DNA hybridization analyses. *E. albertii* infections reported in humans typically describe watery diarrhoea, abdominal pain, dehydration, vomiting and in some cases fever [5]. *E. albertii* has also been isolated from healthy and diseased birds [47]. However, the significance of the epidemiological link of animal reservoir is not fully characterized [23]. *E. albertii* is closely related to *E. coli* based on conventional biochemical characteristics and has been misidentified as EPEC or STEC. Since there have been only a limited number of reports on the isolation and identification of *E. albertii* [48], more studies are needed. In the present study, we report *E. albertii* in paediatric diarrhoeal cases in Kolkata, India. *E*. *albertii* infection seems to be associated more often with children than adults [17] and hence it is important to consider younger age groups for monitoring *E. albertii*-associated infections.

In this study, the prevalence of *E. albertii* was 1.17%, which is very similar to a previous report [49]. Importantly, *E. albertii* has been identified as the sole pathogen. The isolation of *E. albertii* with other pathogens has not been the focus of other studies [5,6]. Clinical presentations caused by *E. albertii* infection were similar to the presentations reported earlier, such as watery diarrhoea, dehydration, fever and abdominal pain [4].

It has been reported that *E. albertii* can be differentiated from other species like *E. fergusonii* and *E. coli* using genetic and/or biochemical markers [50]. In our study, all the *E. albertii* isolates were non-motile and did not ferment lactose. Moreover, all *E. albertii* isolates were positive for *Ea-cdt* gene-specific PCR and *eae* gene. These biochemical and genetic signatures seem useful in the identification of *E. albertii*, which distinguishes the isolates from other closely related bacterial species [23].

The WGS analysis revealed that the virulence genes of the isolates in this study were similar to those reported in other studies [12,14]. *E. albertii* possesses virulence factors, some of which were common with some pathogroups of *E. coli* [2,51]. We found that *E. albertii* strains contain a group of genes encoding intimin, non-LEE effectors, plasmid adhesin, enterotoxin and cytolethal distending toxin. Many of the virulence factors identified in *E. albertii* strains are rarely reported in different pathogroups of *E. coli*, such as ETEC, EAEC and EIEC [47]. There is a need to perform structure-function analysis of the rare virulence genes and investigate their role in the virulence of *E. albertii* using appropriate *in vivo* models of infection [52].

The *E. albertii* isolates belonging to BAPS cluster 2 were found to have a large number of ARGs. In comparison to other isolates within the cluster, the isolates in our study tended to have a lower number of ARGs. However, it is worth noting that one isolate, which belonged to cluster 1, possessed less ARGs within the cluster. Discrepancies between the AST and the WGS analysis suggest that antimicrobial resistance might have been functional through other mechanisms such as unknown efflux pumps or other means.

In our study, we found that in some cases, the isolates were not of animal origin. However, we did not check the food consumption history or information about animal contact to draw conclusions about the likely origins of *E. albertii*. In one study, it was reported that there is a strong association between travel and food consumption in the transmission of *E. albertii* in humans [12]. Furthermore, migratory birds and other avian species may be involved in the spread of *E. albertii*, as *E. albertii* is frequently isolated from birds, including poultry and wild birds [53]. Also, raccoons and other animals have also been reported to harbour *E. albertii* [16]. To better understand the transmission dynamics of *E. albertii* infection, future studies should investigate food consumption, travel history and contact with animals and birds. Exposure to water bodies frequented by migratory birds should also be investigated.

## supplementary material

10.1099/mgen.0.001363 Uncited Supplementary Material 1.

## Abbreviations

ARGs: antimicrobial resistance genes
AST: antimicrobial susceptibility testing
BAPS: Bayesian analysis of population structure
EAEC: enteroaggregative *Escherichia coli*
EHEC: enterohaemorrhagic *Escherichia coli*
EIEC: enteroinvasive *Escherichia coli*
EPEC: enteropathogenic *Escherichia coli*
ETEC: enterotoxigenic *Escherichia coli*
LBA: Luria Bertani agar
LEE: locus of enterocyte effacement
ML: maximum likelihood
MLST: multilocus sequence typing
NCBI: National Center for Biotechnology Information
QRDR: quinolone-resistance determining region
STEC: Shiga toxin-producing *Escherichia coli*
TSI: triple sugar iron
T3SSs: type III secretion systems
WGS: whole-genome sequence
